# Acceleration predicts energy expenditure in a fat, flightless, diving bird

**DOI:** 10.1038/s41598-020-78025-7

**Published:** 2020-12-09

**Authors:** Olivia Hicks, Akiko Kato, Frederic Angelier, Danuta M. Wisniewska, Catherine Hambly, John R. Speakman, Coline Marciau, Yan Ropert-Coudert

**Affiliations:** 1 Centre D’Etudes Biologiques de Chizé, CNRS, La Rochelle Université, UMR 7372, Villiers-en-Bois, France; 2 Institute of Biological and Environmental Sciences, University of Aberdeen, Aberdeen, UK; 3 State Key Laboratory of Molecular Developmental Biology, Institute of Genetics and Developmental Biology, Chinese Academy of Sciences, Beijing, People’s Republic of China

**Keywords:** Ecophysiology, Ecology

## Abstract

Energy drives behaviour and life history decisions, yet it can be hard to measure at fine scales in free-moving animals. Accelerometry has proven a powerful tool to estimate energy expenditure, but requires calibration in the wild. This can be difficult in some environments, or for particular behaviours, and validations have produced equivocal results in some species, particularly air-breathing divers. It is, therefore, important to calibrate accelerometry across different behaviours to understand the most parsimonious way to estimate energy expenditure in free-living conditions. Here, we combine data from miniaturised acceleration loggers on 58 free-living Adélie penguins with doubly labelled water (DLW) measurements of their energy expenditure over several days. Across different behaviours, both in water and on land, dynamic body acceleration was a good predictor of independently measured DLW-derived energy expenditure (R^2^ = 0.72). The most parsimonious model suggested different calibration coefficients are required to predict behaviours on land versus foraging behaviour in water (R^2^ = 0.75). Our results show that accelerometry can be used to reliably estimate energy expenditure in penguins, and we provide calibration equations for estimating metabolic rate across several behaviours in the wild.

## Introduction

Energy is a crucial currency in ecology that can influence behavioural decisions, reproduction and survival and is thus essential to maximising fitness^1–3^. Therefore, measuring energetic costs relative to energy intake is necessary to better understand the mechanistic life-history trade-offs between survival and reproduction^4–6^. However only a few methods have been developed for measuring this important metric on large vertebrates in the wild^7,8^. The heart-rate method can provide high resolution measures of a proxy for energy use but it relies on the physiological relationship between heart rate and $\dot{V}$O_2_, that can be influenced by cardiovascular adjustments occurring irrespective of energy use^8,9^. The standard is the doubly labelled water (DLW) method, which provides a single time-averaged value of energy expenditure^7,10^, although there is some uncertainty as to the efficacy of this technique for diving animals^7^. Another technique uses acceleration^11^ of the body, recorded with animal-attached devices, as a proxy for the energy expended by an individual, based on the concept that energy expended relates to work done^3,12^. One of the most widely used acceleration based proxies is dynamic body acceleration (DBA)^3,13^ which uses the summation of acceleration vectors often measured in all three dimensional axes of the animal’s body^14^, representing the forces acting on the animal's body mass, thereby linking it to work and power^3^. With the improvement and miniaturisation of bio-loggers there has been an increase in studies using accelerometry to estimate energy expenditure in animals^15,16^, however this method must be calibrated against a genuine measure of energy expenditure, such as respirometry or DLW^17^. DBA has been tested against rate of oxygen consumption ($\dot{V}$O_2_) on numerous occasions across taxa in captivity^18–22^ but with more difficulty in the wild^23–27^.

Although many studies have found strong positive correlation between DBA and $\dot{V}$O_2_, there are still uncertainties about how these relationships may vary in the wild, for instance, the relationship between oxygen consumption and mechanical power can vary with muscle power and behavioural mode or medium^28^. Likewise, activities that do not elicit dynamic body acceleration, such as digestion and thermoregulation or ingestion of cold prey, can complicate the relationship between DBA and $\dot{V}$O_2_. Predictive power of DBA for overall power use by an animal decreases as the fraction of non-movement-based power increases^3^. Thermoregulation can be particularly costly and also imposes a proportionately greater metabolic rate on resting than on moving animals because of thermal substitution^29,30^. As well as thermal substitution, diving poses another problem due to the complications of hypometabolism, where oxygen consumption declines exponentially with dive duration^27,31,32^. Furthermore, water loss or gain from diving and ingestion of cold prey^32,33^ can affect the relationship. It is, therefore, no surprise that the two studies in which no relationship was found between $\dot{V}$O_2_ and DBA, were conducted in cold-water homeotherms that operate both in the air and in water^34,35^.

In this context, Antarctic penguins, as flightless birds, provide an interesting model for validating the accelerometry technique in the wild. They live in extremely cold environments, forage by diving to great depths, and spend large periods of time resting on their nests. To test the efficacy of this less invasive technique, here we use it in combination with DLW to answer two questions:

(1) Can accelerometry be used to estimate energy expenditure in free living and diving Adélie penguins *Pygoscelis adeliae*? And if so, (2) do calibration equations differ between behaviours or mediums (air or water)? That is to say, what is the most parsimonious way to estimate overall energy expenditure of wild Adélie penguins using accelerometry?

## Results

**Figure 1:**
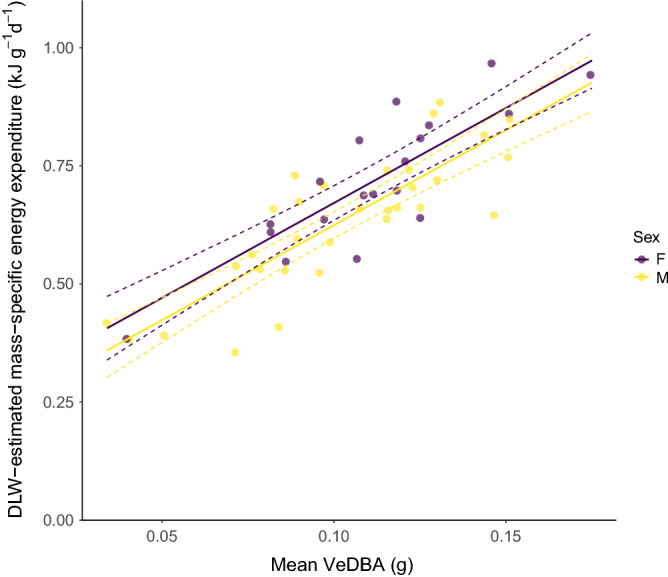
Vectoral dynamic body acceleration (VeDBA) reliably predicts daily energy expenditure (DEE) in Adélie penguins moving freely across different behavioural modes. (model equation: DEE = (0.27 ± 0.05) + (4.02 ± 0.38)_mean_VeDBA + (-0.05 ± 0.02)Sex, *R*^2^ = 0.72, *t*_47_ = 10.705, *p* < 0.0001).

DLW-estimated mass-specific energy expenditure was positively correlated with both measures of DBA (cf. methods): mean vectoral dynamic body acceleration (VeDBA) (*R*^2^ = 0.72, *t*_47_ = 10.705, *p* < 0.0001; Fig. 1) and mean overall dynamic body acceleration (ODBA) (*R*^2^ = 0.72 *t*_47_ = 10.73, *p* < 0.0001). The correlation between total VeDBA and total ODBA was strong (*R*^*2*^ = 0.99, *t*_*48*_ = 366.04, p < 0.0001). VeDBA models performed marginally better than ODBA, though ODBA and VeDBA had very similar predictive abilities of energy expenditure and models were within ΔAIC ≤ 2 of one another. Due to this and the theoretical arguments set out by Qasem et al.^32^, ODBA was omitted from subsequent analysis.

**Figure 2:**
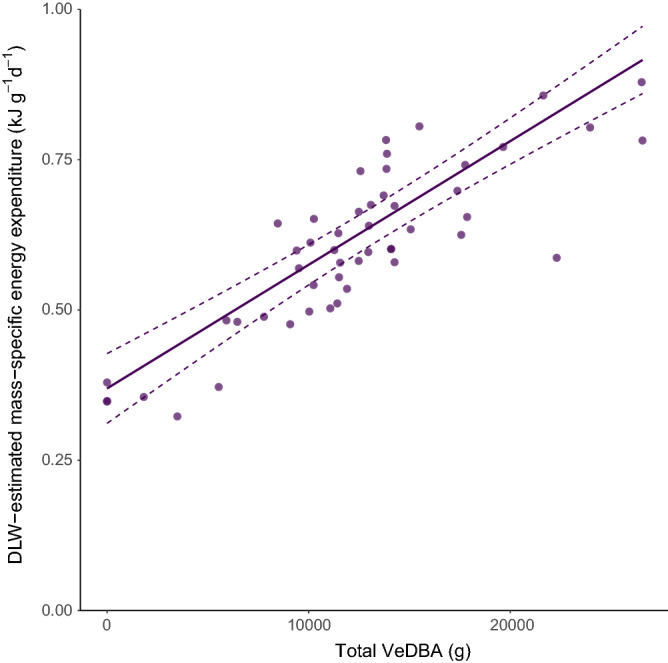
Daily VeDBA for porpoising, diving and surface periods predicts energy expenditure. Raw data points and regression relationship for the significant term in the top model (VeDBA _porpoising, diving and surface_). Full model: DEE ~ VeDBA _porpoising, diving and surface_ + VeDBA_preening_ + VeDBA_land_ + Sex, *R*^2^_=_ 0.75, *p* < 0.0001 (see Eq. 3).

When comparing DBA against time-budget models, DBA was consistently better at explaining daily energy expenditure (DEE) than models using time alone (see Table 1). The best-fitting time-budget model had a **△**AIC of 5 compared to the best VeDBA model. The most parsimonious VeDBA models identified by AIC analysis considered land-based behaviours to have similar coefficients among themselves; and, similarly, at sea behaviours, diving, porpoising and surfacing, as having similar calibration coefficients among themselves (*R*^2^ = 0.75, *p* < 0.001*)* (see Fig. 2 for full model). Within 2 ΔAIC of the best supported model, was an equally supported model assigning just two calibration coefficients, one to all land-based activities, and another to all water-based activities (see Table 1).

**Table 1:** Comparisons among models for explaining energy expenditure in wild Adélie penguins in behavioural modes.

Model parameters	AIC	△ AIC	AIC weight
VeDBA_Porp and Dive and Surface_ + VeDBA_Preen_ + VeDBA_Land_	− 119.41	0.00	0.25
VeDBA_Porp and Dive_ + VeDBA_Surface_ + VeDBA_Preen_ + VeDBA_Land_	− 117.65	1.76	0.10
VeDBA_Porp and Surface_ + VeDBA_Dive_ + VeDBA_Preen_ + VeDBA_Land_	− 117.50	1.91	0.10
VeDBA_Water_ + VeDBA_Land_	− 117.50	1.91	0.10
VeDBA_Porp_ + VeDBA_Preen_ + VeDBA_Dive and Surface_ + VeDBA_Land_	− 117.42	1.99	0.09
*Time_Dive_ + Time_Preen_ + Time_Porp and Surface_ + Time_Land_	− 114.32	5.09	0.02
*Time_LPreen_ + Time_LRest_ + Time_Preen_ + Time_Porp_ + Time_Dive_ + Time_Surface_ + Time_Walk_	− 108.68	10.73	0.00
*VeDBA_all modes_	− 97.18	22.23	0.00

We considered all potential time budget and dynamic body acceleration (DBA) models. We present all models with **Δ**AIC < 2 compared to the best model, as well as three null models: two different time budget models and the model only including average VeDBA across all behavioural modes. Null models are denoted with asterisks.

Sex was not significant in the most parsimonious models (*t*_45_ = 1.539 p = 0.1308).

In the total VeDBA full model (i.e. with all behaviours included), the only variable that was significant in predicting energy expenditure was total VeDBA for diving, responsible for 19% of the variation in energy expenditure.

### Explanatory models

Total VeDBA full model:

1 $$ \begin{aligned} DEE (\mathrm{kj}/\mathrm{g}/\mathrm{d})&= \left(4.76 \times {10}^{-1}\pm 5.218 \times {10}^{-2}\right)+\left(-2.40 \times {10}^{-6}\pm 3.41\times {10}^{-5}\right)VeDB{A}_{LandPreen}\\&\quad+\left(-1.39\times {10}^{-5}\pm 1.03\times{10}^{-5}\right)VeDB{A}_{Rest}  +\left(-3.70\times {10}^{-5}\pm 3.02\times{10}^{-5}\right)VeDB{A}_{Preen} \\ \\&\quad+\left(2.85\times {10}^{-5}\pm 2.27\times{10}^{-5}\right)VeDB{A}_{Porpoise}+\left(2.17\times {10}^{-5}\pm 3.94\times{10}^{-6}\right)VeDB{A}_{Dive} \\&\quad +\left(1.28\times {10}^{-5}\pm 1.42\times{10}^{-5}\right)VeDB{A}_{SeaSurface} +\left(-9.38\times {10}^{-6}\pm 9.37\times{10}^{-6}\right)VeDB{A}_{Walk}\\&\quad+\left(-3.22\times {10}^{-2}\pm 2.34\times {10}^{-2}\right)Sex \end{aligned} $$

Total ODBA full model:

2 $$ \begin{aligned}DEE &=\left(4.75 \times {10}^{-1}\pm 5.26 \times {10}^{-2}\right) +\left(-2.57\times {10}^{-6}\pm 2.43\times {10}^{-5}\right)ODB{A}_{LandPreen}\\&\quad+\left(-9.26\times {10}^{-6}\pm 7.1\times {10}^{-6}\right)ODB{A}_{Rest}+\left(-2.75\times {10}^{-5}\pm 2.18\times {10}^{-5}\right)ODB{A}_{Preen}\\&\quad+\left(2.40\times {10}^{-5}\pm 1.61\times {10}^{-5}\right)ODB{A}_{Porpoise}+\left(1.49\times {10}^{-5}\pm 2.76\times {10}^{-6}\right)ODB{A}_{Dive}\\&\quad+\left(8.87\times {10}^{-6}\pm 9.98\times {10}^{-6}\right)ODB{A}_{SeaSurface}+\left(-6.59\times {10}^{-6}\pm 6.65\times {10}^{-6}\right)ODB{A}_{Walk}\\&\quad+\left(-3.36\times {10}^{-2}\pm 2.34\times {10}^{-2}\right)Sex\end{aligned} $$

Total VeDBA most parsimonious model:

3 $$ \begin{aligned}DEE&=\left(4.64 \times {10}^{-1}\pm 54.01 \times {10}^{-2}\right) +\left(-3.46 \times {10}^{-5}\pm 2.83\times {10}^{-5}\right)VeDB{A}_{Preen}\\&\quad+\left(2.06\times {10}^{-5}\pm 1.84\times {10}^{-6}\right)VeDB{A}_{Porpoise+Dive+SeaSurface}\\&\quad+\left(-1.05\times {10}^{-5}\pm 5.15\times {10}^{-6}\right)VeDB{A}_{LandPreen+Rest+Walk}\\&\quad+\left(-3.14\times {10}^{-2}\pm 2.04\times {10}^{-2}\right)Sex\end{aligned} $$

Total VeDBA comparable model (within 2 AIC of the best supported model):

4 $$ \begin{aligned}DEE&=\left(4.54 \times {10}^{-1}\pm 4.09 \times {10}^{-2}\right)+\left(1.93\times {10}^{-5}\pm 1.76\times {10}^{-6}\right)VeDB{A}_{Water}\\&\quad+\left(-1.16\times {10}^{-5}\pm 5.25\times {10}^{-6}\right)VeDB{A}_{Land}+\left(-3.08\times {10}^{-2}\pm 2.09\times {10}^{-2}\right)Sex\end{aligned} $$

Best supported time-budget model:

5 $$ \begin{aligned}DEE &=\left(-0.45\pm 0.12\right){Time}_{Dive} +\left(-0.07\pm 0.02\right){Time}_{Preen} \\&\quad+\left(-0.11\pm 10.07\right){Time}_{Porpoise+SeaSurface} \\&\quad+\left(-0.67\pm 0.17\right){Time}_{Land} +\left(-0.09\pm 0.18\right)Sex\end{aligned} $$

## Discussion

We show that dynamic body acceleration predicts energy expenditure, measured via doubly labelled water, in free-living birds inhabiting an extreme environment, and across several behavioural modes including prolonged diving in sub-zero water. Thus, despite the confounding effects of variables such as thermoregulation, digestion and variability in muscle efficiency between different media, accelerometry provides a reliable index of overall energy expenditure. Group error associated with the doubly labelled water technique is low (0.5%)^37^ however, individual level variation with this method is greater (~ 10%)^10^, which is likely to reduce the strength of calibration correlation with DBA. Therefore, accelerometry may be a better measure of energy expenditure than our analyses suggest.

For the duration of the study (Dec 2018–Jan 2019) foraging trips were particularly short due to above-average foraging conditions, meaning that a larger proportion of the experiment was spent in inactive resting behaviours as opposed to active foraging behaviour. This may be another consideration when estimating field metabolic rates between years and conditions and could mean that in poorer years, where foraging trips are longer^38^, the relationship could be better between overall DBA and DLW. These shorter foraging trips can also be demonstrated by comparing the range of field metabolic rates (FMR) from this study (1450–4440 kJ/d), to those previously estimated for foraging Adélies (2412 to 6915 kJ/d)^39,40^. Although our estimates are within a similar range, they are on average lower than previously estimated values. Likewise, the power values previously recorded for Adélies’ resting metabolic rates are 6.9, 11 and 8.4 W/kg^41,42^ and in this study we calculate a power value of 6.9 W/kg. The intercept of the model predicting energy expenditure with mean VeDBA shows basal mass-specific metabolic rate to be around 270 kJ/kg/d. This is similar to the mean rate of 267 kJ/kg/d in^40^ suggesting that our estimates of resting metabolic rate are realistic and similar to previous studies.

Using accelerometry with DLW not only allows the calibration of overall energy expenditure but the understanding of how well DBA predicts energy expenditure across different behaviours. We found that different behaviours have different calibration coefficients, largely split between land and water, however diving and porpoising and water surface behaviour all had the same calibration coefficient (see Eq. 3). We provide behaviour-specific calibration coefficients for DBA, which can be used to calculate energy expenditure from simple time budgets (Eq. 5) based on diving data alone, as well as acceleration data.

Air-breathing divers have produced equivocal results when metabolic rate was measured, and as such in these studies DBA was found to correlate weakly or not at all with metabolic rate^23,34^. Yet the R^2^ in this study in the wild (0.72) is higher than values reported for other vertebrates measured in captivity/semi-captivity (0.47 for diving Steller sea lions *Eumetopias jubatus*^43^; 0.60 for swimming sharks^44^; and 0.56 for turtles^45^). Although the proportion of variation in energy expenditure explained by DBA is high, it is a little lower than in some other studies of avian diving species, such as cormorant and murre^25,27,46^. This is likely due to a large proportion of the time spent in the water with heat loss being incorporated into the measurement of the energy expenditure by DLW but not by DBA. In volant species, strong relationships are often driven by the high variation in DBA and energy expenditure in flight, thus in penguins where this is not the case, DBA and energy expenditure relationships may be less strong.

Animals are known to incur different energetic costs to undertake different activities, and different gradients of relationships are known to exist between VeDBA and energy expenditure depending on gaits in humans^47^, intensity of swimming in sharks^48^ and the muscles involved in the movement of cormorants^49^. Indeed we found the relationship between DLW and DBA to differ in different behaviours, with the largest differences occurring between land- and water-based behaviours. This is expected due to the difference in medium and also the mechanics of the muscles used, and similar results were found in other air-breathing diving species^25,50,51^. Although preening and walking have the highest mean DBA, diving contributes the most to FMR due to the proportion of time spent foraging at sea. We found DBA whilst foraging at-sea provides a very good calibration relationship with overall daily energy expenditure (see Eq. 3 and Fig. 2.), despite diving DBA often being a poor proxy for energy expenditure due to the restriction of heart rate^17^ and specific heat loss^52,53^. As penguins are adapted to diving in cold waters, they are likely to have lower costs of diving than diving volant species due to reduced heat loss. This is likely to contribute to the strong relationship we find between foraging at-sea DBA and energy expenditure. Additionally, the most parsimonious model includes whole diving bouts, thus surface periods and diving both have the same correlation coefficient as in^24,43^, meaning that the oxygen replenishment between each dive is included in the measurement.

Despite the strong relationship between DBA and metabolic rate during active foraging, penguins spend a large amount of time inactive on land, and, as seen in previous calibrations the relationship between DBA and metabolic rate decreases during low activity behaviours^54^. Additionally, in such cold environments inactivity can incur high thermoregulatory costs which are expressed in the DLW measurement but not DBA.

In conclusion, we show that DBA is an effective measure of energy expenditure in a marine predator living in extreme conditions. Behaviour-specific metabolic rates can be estimated in free-ranging conditions over multiple media, meaning our calibration equations can be used to estimate behaviour-specific or total daily energy expenditure by deploying small accelerometers or time-depth recorders on Adélie penguins and probably other Pygoscelid penguins, like Chinstrap *P. antarcticus* and Gentoo *P. papua*, although the latter typically utilizes warmer temperature waters. The advantage of this calibration is that it was estimated over several days with a relatively large sample size, meaning the estimates are likely to be more representative and accurate than short-term calibrations^8^. Yet, using an accelerometry technique allows for both long- and short-term energy expenditure to be estimated in the wild^17^, as well as for relatively fine-scale behaviours, such as diving or preening. In particular, as previous studies of diving metabolic rate have produced equivocal results, by subdividing at-sea activity into separate behaviours, we were able to examine the differences in metabolic rate of diving and consequent surface time. Furthermore, this study calibrated accelerometry in the natural environment in which future uses of the estimates are most likely to be applied.

## Methods

### Data collection procedure

We captured 58 adult Adélie penguins, 24 females and 34 males, in the colony on Ile des Pétrels, where the Dumont d’Urville station is also found, in Terre Adélie, East Antarctica (66°40′ S; 140°01′ E), between 21st December 2018 and 14th January 2019. All birds were breeders during the chick guarding stage, in which one parent guards the chicks on the nest while the other is foraging at sea to bring food back to its offspring. Individuals were captured on the nest when both adults were attending the nest prior to a changeover, though only one member of a pair was ever used for the experiment, to reduce disturbance time to the nest.

Daily energy expenditure (DEE, kJ/g/day) was measured using the DLW technique^7,55^. This method has been previously validated by comparison to indirect calorimetry in a range of small mammals (e.g.^56^). Upon capture, individuals were blood sampled from the tarsus vein to take a background sample, then weighed to the nearest gram and injected with 0.3 ml of DLW per kg of body weight into the pectoral muscle (enrichment H_2_^18^O 653,405 ppm and D_2_O 342,560 ppm, Speakman Lab). Syringes were weighed before and after administration (± 0.0001 g) to calculate the exact amount of DLW injected, and the time of injection recorded. For future identification, the birds were marked with a unique identifying code printed on a piece of marine tape rolled around their back feathers, and then placed in a contained area outside the lab for the DLW to equilibrate (for between 1.6 and 2.7 h). Individuals were then fitted with accelerometers (see below) and a second blood sample was taken (the initial) from the tarsus vein. The birds were released back to their nests and kept under distant observation until they were seen departing for a foraging trip at sea. Either when an individual returned from foraging, or 3 days after DLW injection, the bird was recaptured from the nest and the time recorded. If the individual was the only parent present at the nest, chicks were kept warm and safe whilst the adult was processed. A blood sample was taken and the bird was weighed. The bird was returned to its nest and we ensured that it continued chick guarding and feeding for several days (in no instance were study nests abandoned). Mean bird handling time was < 15 min (including DLW injection, blood samples before and after DLW equilibrium period and logger attachment). Additionally three non-breeding control group individuals were captured and processed as above with DLW and equipped with an accelerometer but kept in a contained area outside for the duration of the experiment (two days). Molecular sexing was performed to confirm the sex of each individual a posteriori^57^.

Comité d'Ethique en Expérimentation Animale Numéro 084, Terres Australes et Antarctiques Françaises, Comité d'Environnement Polaire and Conseil National de la Protection de la Nature all approved this experiment to be carried out and all experiments were performed in accordance with these guidelines and regulations.

### Doubly labelled water measurements

Blood samples were immediately transferred to glass capillary tubes, heat sealed and stored at room temperature until distillation could be carried out. Analysis of the isotopic enrichment of blood was performed blind, using a Liquid Isotope Water Analyser (Los Gatos Research, USA)^58^. Initially, the blood encapsulated in capillaries was vacuum distilled^59^, and the resulting distillate was used for analysis. Samples were run alongside five lab standards for each isotope and international standards, to correct delta values to ppm. A single-pool model was used to calculate rates of CO_2_ production as recommended for use in animals around 5 kg in body mass^60^. There are several approaches for the treatment of evaporative water loss in the calculation^61^. We assumed evaporation of 25% of the water flux (eq. 7.17^10^) which minimizes error in a range of conditions^10,61,62^. A respiratory quotient of 0.8 was used in the calculations, representing a diet of fish and krill^40,63^.

### Accelerometry and Behavioural assignment

Data-loggers (Axy-Trek, Technosmart, Italy, 40 × 20 × 8 mm, 14 g) were attached to the central dorsal region of the bird with waterproof adhesive Tesa tape^64^, and secured with two Colson plastic cable ties. The loggers sampled tri-axial acceleration at 100 Hz and pressure at 1 Hz data were sampled continuously during the deployment. Upon recovery, data were processed using custom written scripts in Igor Pro (version 8.03, Wavemetrics, USA). Depth was calculated from pressure, ﻿DBA was calculated by smoothing data for each axis across a 1-s period to calculate the static acceleration, and then subtracting the static acceleration from the raw acceleration values. ODBA is the sum of the absolute dynamic body acceleration of the three axes, whereas VeDBA is the square root of the sum of the squares of dynamic body acceleration in the three axes^36^:

$$VeDBA= \sqrt{{A}_{X}^{2}+{A}_{Y}^{2}{+A}_{Z}^{2}}$$

$$ODBA= {|A}_{X}|+|{A}_{Y}|+\left|{A}_{Z}\right|$$

where Ax, Ay and Az are the derived dynamic accelerations at any point in time corresponding to the three orthogonal axes of the accelerometer.

Behaviours were categorised using accelerometer data to differentiate between seven main activities both on land (walking, resting, preening), and in water (diving, porpoising, sea surface and preening). Discrimination of behaviours was done using a histogram segregation method employed in Collins et al.^65^ and Patterson et al.^66^.

Roll and pitch, measures of body rotation and angle were calculated using the following equations:

$${\mathrm{Pitch}}={\mathrm{arctan}}\left(\frac{X}{{\left({Y}^{2}+{Z}^{2}\right)}^\frac{1}{2}}\right)*\left(\frac{180}{\pi }\right)$$

$${\mathrm{Roll}}={\mathrm{arctan}}\left(\frac{Y}{{\left({X}^{2}+{Z}^{2}\right)}^\frac{1}{2}}\right)*\left(\frac{180}{\pi }\right)$$

where X is acceleration in the surge axis, Y is acceleration in the sway axis, and Z is acceleration in the heave axis.

Diving was differentiated from all other behaviours using a pre-existing macro in Igor Pro with a depth threshold of 1 m. Time spent at sea was divided into diving and surface time where periods of diving to depths of less than 2 m were classified as porpoising. Likewise, surface time was categorised as preening when periods were above a threshold of standard deviation of roll (mean breakpoint 15), determined with the histogram segregation method. Within the period spent on land, walking was assigned using both pitch (mean breakpoint 60°) and mean VeDBA (mean breakpoint 0.25). Resting on land was separated from preening on land using mean VeDBA (mean breakpoint 0.1).

For all individuals, we calculated the proportion of time spent in each behaviour (time budget) and the mean VeDBA and ODBA value for each behaviour. We also calculated total VeDBA and ODBA, as the mean behavioural DBA value multiplied by the duration of time spent in that behaviour per day.

### Statistical analysis

To address question 1: can accelerometry be used to estimate energy expenditure? We modelled DLW-derived estimates of energy expenditure using general linear models with sex and total VeDBA or total ODBA as explanatory variables. To address question 2: what is the most parsimonious way to predict energy expenditure with accelerometry? We compared models predicting energy expenditure by the proportion of time spent in different behaviours (i.e. a time budget) against those predicting energy expenditure with total VeDBA or total ODBA for different behaviours. We used multiple regressions as each activity is likely to have different costs and DBA-to-metabolic-rate relationships. Time-budget models used log-ratios of the proportion of time spent in behaviours to predict energy expenditure. These models had an intercept forced to zero, as when no time passes no energy is expended^25,27^. We compared models with separate calibration coefficients for each behavioural mode to models that had a single calibration coefficient that applied across all behavioural modes. Sex was included as a variable in all models to account for any sex differences in metabolic rate relationships. In all models mass was accounted for in measurements of DLW-derived estimates of energy expenditure (DEE kJ/g/d) see supplementary materials table S1 for mass of all study individuals. We estimated calibration coefficients that minimized the log-likelihood of a particular general linear model describing daily energy expenditure (dependent variable) from dynamic acceleration or time (independent variables). We compared all combinations of time budget and VeDBA models using an Akaike’s information criterion (AIC) approach which penalises models with a larger number of parameters but no improvement of fit. All statistical analyses were conducted in R version 3.6.2^67^.

## Supplementary information

Supplementary information
