# Supplementary material for: Acceleration predicts energy expenditure in a fat, flightless, diving bird
Source: Sci Rep. 2020 Dec 9;10:21493. doi: 10.1038/s41598-020-78025-7 (PMC7726140; doi:10.1038/s41598-020-78025-7)
Supplement: Supplementary file 1 — Supplementary information [file 41598_2020_78025_MOESM1_ESM.docx]

**Supplementary information**

**Acceleration predicts energy expenditure in a fat, flightless, diving bird**

Olivia Hicks^1*^, Akiko Kato^1^, Frederic Angelier^1^, Danuta M. Wisniewska^1^, Catherine Hambly^2^, John R. Speakman^2,3^, Coline Marciau^1^, Yan Ropert-Coudert^1^

^1^Centre d’Etudes Biologiques de Chizé, CNRS, La Rochelle Université, UMR 7372, Villiers-en-Bois, France

^2^ Institute of Biological and Environmental Sciences, University of Aberdeen, Aberdeen, UK

^3^ State Key Laboratory of Molecular Developmental Biology, Institute of Genetics and Developmental Biology, Chinese Academy of Sciences, Beijing, People's Republic of China

***** Correspondence to olivia.c.hicks@gmail.com

**Table S1 :** Behavioural information for study individuals. Mean duration per day of each behavioural activity is presented with mass and sex.

| **ID** | **Sex** | **Mass (g)** | **Dive (h)** | **Rest (h)** | **Float (h)** | **Walk (h)** | **Porpoise (h)** | **Preen (h)** |
| --- | --- | --- | --- | --- | --- | --- | --- | --- |
| F1 | M | 5280 | 2.64 | 19.44 | 0.04 | 0.49 | 0.92 | 0.74 |
| F2 | F | 4556 | 5.16 | 15.30 | 2.10 | 0.58 | 0.10 | 0.24 |
| F3 | F | 5051 | 8.57 | 10.96 | 2.40 | 0.67 | 0.91 | 0.23 |
| F5 | F | 5145 | 3.77 | 17.86 | 0.76 | 0.85 | 0.49 | 0.08 |
| F6 | M | 4869 | 3.12 | 18.65 | 0.96 | 0.88 | 0.08 | 0.19 |
| F7 | M | 5466 | 3.50 | 18.62 | 0.91 | 0.56 | 0.11 | 0.15 |
| F8 | M | 5278 | 2.53 | 18.80 | 0.94 | 1.29 | 0.05 | 0.04 |
| F9 | M | 5487 | 0.42 | 22.13 | 0.32 | 0.57 | 0.12 | 0.07 |
| F11 | F | 4902 | 8.36 | 12.58 | 1.96 | 0.62 | 0.03 | 0.34 |
| F12 | M | 5844 | 8.12 | 12.06 | 1.83 | 1.44 | 0.14 | 0.25 |
| F13 | F | 4310 | 6.66 | 13.08 | 3.07 | 0.34 | 0.41 | 0.18 |
| F14 | M | 4987 | 8.09 | 12.45 | 2.21 | 0.86 | 0.09 | 0.22 |
| F15 | M | 5108 | 4.95 | 17.15 | 1.11 | 0.42 | 0.13 | 0.11 |
| F16 | F | 4462 | 6.33 | 14.27 | 1.61 | 0.77 | 0.79 | 0.12 |
| F18 | F | 4132 | 5.11 | 16.68 | 1.44 | 0.42 | 0.11 | 0.08 |
| F19 | M | 4875 | 8.17 | 12.48 | 2.03 | 0.79 | 0.21 | 0.15 |
| F20 | M | 5284 | 7.01 | 14.02 | 1.77 | 0.74 | 0.12 | 0.13 |
| F21 | M | 4396 | 6.27 | 15.23 | 1.63 | 0.53 | 0.03 | 0.18 |
| F22 | M | 4486 | 6.97 | 13.88 | 1.91 | 0.53 | 0.44 | 0.13 |
| F24 | M | 4184 | 8.47 | 10.97 | 2.26 | 0.95 | 1.03 | 0.30 |
| F25 | M | 4986 | 2.21 | 18.80 | 0.75 | 1.69 | 0.05 | 0.18 |
| F26 | F | 5036 | 4.33 | 16.45 | 1.61 | 0.61 | 0.77 | 0.06 |
| F27 | F | 4557 | 8.74 | 10.14 | 3.05 | 0.93 | 0.85 | 0.11 |
| F29 | M | 5219 | 5.69 | 14.53 | 2.06 | 0.99 | 0.49 | 0.15 |
| F30 | M | 4378 | 3.64 | 17.86 | 1.16 | 0.76 | 0.41 | 0.08 |
| F31 | M | 4622 | 5.19 | 14.63 | 2.09 | 1.30 | 0.44 | 0.16 |
| F32 | M | 4824 | 4.15 | 17.58 | 1.29 | 0.46 | 0.19 | 0.18 |
| F33 | M | 4411 | 8.39 | 11.17 | 2.92 | 0.77 | 0.62 | 0.11 |
| F34 | F | 4604 | 7.14 | 12.82 | 2.41 | 0.72 | 0.71 | 0.08 |
| F35 | M | 4750 | 6.54 | 14.37 | 1.97 | 0.67 | 0.23 | 0.18 |
| F36 | M | 4691 | 5.18 | 16.54 | 1.41 | 0.50 | 0.11 | 0.19 |
| F38 | M | 4733 | 3.60 | 17.50 | 1.29 | 0.89 | 0.33 | 0.13 |
| F39 | M | 4293 | 5.38 | 16.07 | 1.62 | 0.57 | 0.11 | 0.09 |
| F40 | M | 4597 | 8.03 | 10.85 | 2.83 | 1.35 | 0.77 | 0.10 |
| F41 | F | 4224 | 3.24 | 18.30 | 0.89 | 0.73 | 0.23 | 0.11 |
| F42 | F | 4441 | 4.77 | 16.54 | 1.38 | 0.54 | 0.43 | 0.14 |
| F43 | F | 4116 | 5.16 | 16.83 | 1.34 | 0.26 | 0.11 | 0.09 |
| F44 | F | 4385 | 6.22 | 15.15 | 1.70 | 0.56 | 0.20 | 0.11 |
| F46 | M | 4847 | 4.37 | 17.40 | 1.37 | 0.40 | 0.13 | 0.04 |
| F48 | M | 4066 | 5.98 | 15.72 | 1.08 | 1.10 | 0.03 | 0.04 |
| F49 | F | 3951 | 5.69 | 14.56 | 2.04 | 1.25 | 0.10 | 0.16 |
| F50 | F | 4372 | 7.07 | 13.47 | 2.05 | 0.92 | 0.22 | 0.17 |
| F51 | M | 4791 | 7.48 | 12.36 | 2.84 | 0.52 | 0.39 | 0.13 |
| F52 | M | 4590 | 5.81 | 14.78 | 1.74 | 1.09 | 0.25 | 0.07 |
| F53 | M | 4674 | 6.34 | 13.44 | 2.30 | 1.02 | 0.75 | 0.06 |
| F54 | F | 4624 | 4.55 | 16.27 | 1.62 | 0.81 | 0.35 | 0.15 |
| F55 | F | 3917 | 7.46 | 14.02 | 1.63 | 0.56 | 0.11 | 0.08 |
